# Discovery of the inhibitor of DNA binding 1 as a novel marker for radioresistance in pancreatic cancer using genome-wide RNA-seq

**DOI:** 10.20517/cdr.2022.60

**Published:** 2022-10-18

**Authors:** Oscar Zuniga, Stephanie Byrum, Adam R. Wolfe

**Affiliations:** ^1^Department of Radiation Oncology, University of Arkansas for Medical Sciences, Little Rock, AR 72205, USA.; ^2^Department of Biochemistry and Molecular Biology, University of Arkansas for Medical Sciences, Little Rock, AR 72205, USA.

**Keywords:** Pancreatic cancer, radiation resistance, ID1, RNA-seq

## Abstract

**Purpose/Objective(s):** Discovery of genetic drivers of radioresistance is critical for developing novel therapeutic strategies to combine with radiotherapy of radioresistant PDAC. In this study, we used genome-wide RNA-seq to identify genes upregulated in generated radioresistant PDAC cell lines and discovered the Inhibitor of DNA Binding 1 (ID1) gene as a potential regulator of radioresistance in PDAC.

**Materials/Methods:** Radioresistant clones of the PDAC cell lines MIA PaCa-2 and PANC-1 were generated by delivering daily ionizing irradiation (IR) (2 Gy/day) *in vitro* over two weeks (total 20 Gy) followed by standard clonogenic assays following one week from the end of IR. The generated RR and parental cell lines were submitted for RNA-seq analysis to identify differentially expressed genes. The Limma R package was used to calculate differential expression among genes. Log2 fold change values were calculated for each sample compared to the control. Genes with an absolute fold change > 1 were considered significant. RNA sequencing expression data from the Cancer Genome Atlas (TCGA) database was analyzed through the online databases GEPIA, cBioPortal, and the Human Protein Atlas.

**Results:** Following exposure to two weeks of 2 Gy daily IR in vitro, the two PDAC cell lines showed significantly greater clonogenic cell survival than their parental cell lines, indicating enhanced RR in these cells. RNA-seq analysis comparing parental and RR cell lines found upregulated seven genes (TNS4, ZDHHC8P1, APLNR, AQP3, SPP1, ID1, ID2) and seven genes downregulated (PTX3, ITGB2, EPS8L1, ALDH1L2, KCNT2, ARHGAP9, IFI16) in both RR cell lines. Western blotting confirmed increased expression of the ID1 protein in the RR cell lines compared to their parental cell lines. We found that ID1 mRNA was significantly higher in PDAC tumors compared to matched normal and high ID1 expression correlated with significantly worse disease-free survival (DFS) in PDAC patients (HR = 2.2, log rank *P *= 0.009). ID1 mRNA expression was also strongly correlated in tumors with TP53 mutation, a known driver of radioresistance.

**Conclusion:** Our analysis indicates a novel role of ID1 in PDAC radioresistance. ID1 expression is higher in tumor tissue compared to normal, and high expression correlates with both worse DFS and association with the TP53 mutation, suggesting that targeting ID1 prior to IR is an attractive strategy for overcoming radioresistance in PDAC.

## INTRODUCTION

Pancreatic Ductal Adenocarcinoma (PDAC) is one of the most lethal solid tumors with a rapid progression course and poor prognosis, contributing to a 10% of five-year survival rate for all stages^[1]^. By the year 2040, the incidence of PDAC is expected to increase from 56,000 to 96,000 cases, a 66% increase. Pancreatic cancer is projected to become the second leading cause of cancer-related death, killing ~46,000 patients yearly in the U.S. alone^[2]^. The only hope for long-term survival in patients with localized PDAC is surgical resection of the tumor. However, upwards of 60% of the patients with potentially curable localized PDAC do not present with frank eligibility for surgical resection^[3]^, and therefore require an aggressive therapy regimen to reduce tumor burden enough to elicit removal. The standard course includes 3-6 months of multi-regimen genotoxic chemotherapies and, commonly, an additional 3-6 weeks of radiation therapy (RT). For most locally advanced PDAC patients, curative surgery will not be achieved despite these aggressive therapies due to the poor response of the tumor and thus remain unresectable. The rate of conversion from unresectable to resectable disease remains at a dismal ~15%^[4]^. Unresected patients have nearly the same dismal survival rates as metastatic disease, with a prognosis of roughly 15-16 months and a poor quality of life^[5]^. Why PDAC responds so poorly to typically effective strategies in other cancers is not entirely clear. It is speculated that the natural prevalence of robust DNA repair mechanisms in PDAC underlies resistance to genotoxic therapy^[6]^. Therefore, understanding the biological underpinnings of radioresistant systems in PDAC is critical for uncovering vulnerabilities in the resistant phenotype.

In this study, we generated radiation-resistant (RR) PDAC cell lines *in vitro* by treating them with daily doses of ionizing radiation and allowing the cells to recover, followed by an analysis of the changes in gene expression. Through next-generation RNA sequencing (RNA-seq), we discovered several novel genes differentially expressed after RR, including the inhibitor of DNA binding-1 (ID1). The ID1 gene has been previously shown to be upregulated in pancreatic cancer cells following nicotine stimulation via a Src kinase-dependent fashion leading to chemoresistance to gemcitabine treatment. Furthermore, elevated ID1 was shown to correlate with worse overall survival in resected pancreatic cancer patients^[7]^. Another study in pancreatic cancer showed ID1 uncouples TGFβ-induced EMT from apoptosis leading to enhanced cell survival^[8]^. The current study validates ID1 as a marker for radiation resistance in PDAC cells for the first time.

## METHODS

### Cell culture and materials

The human pancreatic adenocarcinoma cell lines MIA PaCa-2 and PANC1 were obtained from American Type Culture Collection (Manassas, VA) and maintained at 37 °C in 5% CO_2_ in DMEM media and supplemented with 10% fetal bovine serum (Hyclone, Logan, UT) and penicillin/streptomycin (Life Technologies, Grand Island, NY).

### Generation of radioresistant PDAC cell lines

Cells were subjected to 2 Gray (Gy) radiation daily for 5 days, and then allowed to recover for two weeks, followed by a second course of radiation of 2 Gy daily for 5 days for a total of 20 Gy. Throughout the irradiation process and recovery time, cells were kept at 40%-70% confluency to ensure the potential for exponential growth. After completion of the second week of radiation, cells were collected for protein lysates, plated as single cells for clonogenic assays, or submitted for whole genome sequencing. Radioresistance of the cell lines was verified by comparing the radiosensitivity of the radiation-selected cells (after a recovery period) with their respective parental cell lines by the clonogenic assay as described below.

### Immunoblotting

Cell lysates were prepared using RIPA buffer (ThermoFisher, Waltham MA) supplemented with 1x protease (Complete, Roche, Indianapolis, IN) and phosphatase inhibitors (PhosSTOP, Roche, Indianapolis, IN, Roche), followed by protein quantification by the Dc protein assay kit (Bio-Rad, Hercules, CA). Equal amounts of protein were loaded and resolved by SDS/PAGE and transferred to nitrocellulose membranes. The ID1 (Santa Cruz sc-133104) and GAPDH (Cell Signaling D16H11) primary antibodies were allowed to bind overnight at 4 °C and used at a dilution of 1:100-1000. After washing in TBS-Tween, membranes were incubated with StarBright Goat Anti-Mouse/Rabbit IgG secondary antibodies (Bio-Rad) diluted 1:2500-1:5000 for 1 h. Membranes were washed with TBS-Tween and then imaged on the ChemiDoc MP Imaging System (Bio-Rad).

### Radiation clonogenic assays

Cells were trypsinized to generate single cell suspensions and seeded onto 60 mm tissue culture plates in triplicate. Cells were then irradiated with various doses (0-8 Gy). Ten to 14 days after seeding, colonies were fixed with Methanol/Acetic Acid and stained with 0.5% crystal violet, and the numbers of colonies or colony forming units (CFU) containing at least 50 cells were counted using a dissecting microscope (Leica Microsystems, Inc. Buffalo Grove, IL) and surviving fractions calculated. Experiments were repeated multiple, independent times.

### Experimental radiation

Irradiation was performed with the X-ray at 160 kV, 25 mA at a dose rate of approximately 113 cGy/min using an X-RAD 320 Biological Irradiator (Precision X-Ray Inc.).

### RNA library construction and sequencing

Total RNA was isolated with the TRIzol Reagent protocol (ThermoFisher, Waltham, MA) from the parental and RR cell lines. RNA quality and concentration were estimated with Bioanalyzer 2100 and RNA 6000 Nano Kit (Agilent Technologies, Waldbronn, Germany). RNA-Seq libraries were prepared with Illumina TruSeq Stranded mRNA LT Sample Preparation Kit (Illumina, San Diego, CA) using 1 μg of total RNA according to the manufacturer’s protocol. Sequencing was performed by the genomics core at UAMS on the NovaSeq 6000 platform (Illumina).

### RNA-seq analysis

RNA-seq reads were quality-checked, trimmed, and aligned to the hg38 reference genome (accession: GCA_000001405.15) using the Nextflow RNAseq pipeline, nf-core/rnaseq (version 3.4) available at DOI 10.5281/zenodo.1400710. The resulting gene counts were transformed to log2 counts per million (CPM)^[9]^. Lowly expressed genes were filtered out and libraries were normalized by the trimmed mean of M-values^[10]^. The Limma R package was used to calculate differential expression among genes^[11]^. Log2 fold change values were calculated for each sample compared to the control. Genes with an absolute fold change > 1 were considered significant. 

### Human patient data

The following websites were utilized to query for tumor and normal expression and clinical data for patients with PDAC in the TCGA: https://cbioportal.org/^[12]^, http://gepia.cancer-pku.cn/^[13]^, and https://www.proteinatlas.org/^[14]^.

### Statistical analysis

For *in vitro* experiments, data are presented as the mean ± SEM for clonogenic survival. Statistical comparisons were made between the control and experimental conditions using the two-sided two-group *t*-tests with significance assessed at *P* < 0.05.

## RESULTS

### Generation of radioresistant cell lines

**Figure 1:**
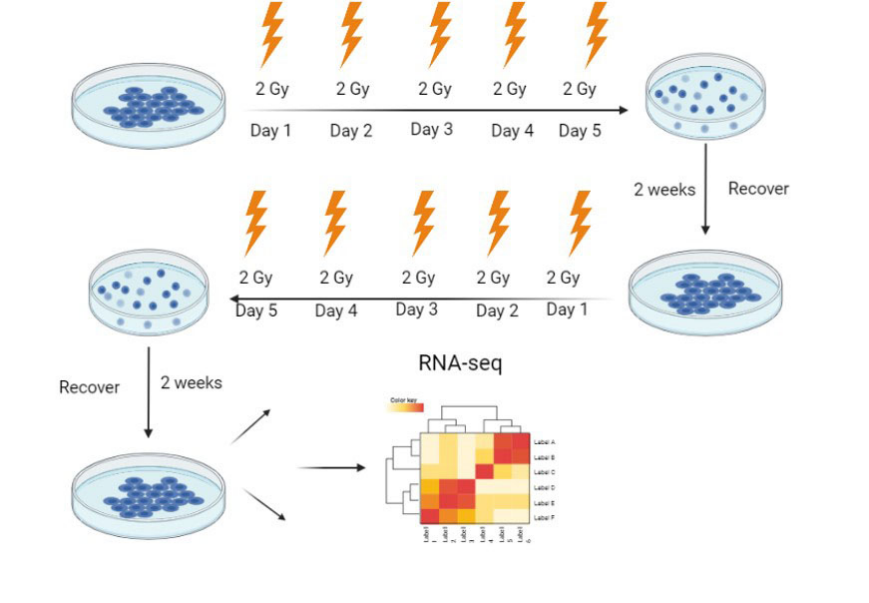
Generation of radioresistant (RR) PDAC cell lines. Schematic of the generation of RR sublines. Cell lines were exposed to 5 fractions of 2 Gy irradiation over one week and then allowed to recover for two weeks, followed by a second course of 5 fractions of 2 Gy over one week and an additional 2-week recovery period. Subsequently, RR cells were collected for RNA-seq analysis, western blotting, and sensitivity to radiation using the clonogenic assay to compare the parental and RR sublines and created with BioRender.com.

To generate RR cell lines, we delivered 10 Gy of IR (2 Gy/day) to PDAC MIA PACA-2 and PANC-1 cells *in vitro *over five days, followed by a recovery time of 2 weeks followed by a second course of 10 Gy in 2 Gy per day fractions followed by an additional two weeks of recovery [Figure 1]. In parallel, we cultured the parental cells and exposed them to 10 daily doses of sham radiation. Following the generation of RR cells, we performed radiation clonogenic assays comparing the parental and RR cells. In addition, we performed whole-genome sequencing on the parental controls and RR cells in triplicate using the next-generation NovaSeq 6000 RNA sequencer.

**Figure 2:**
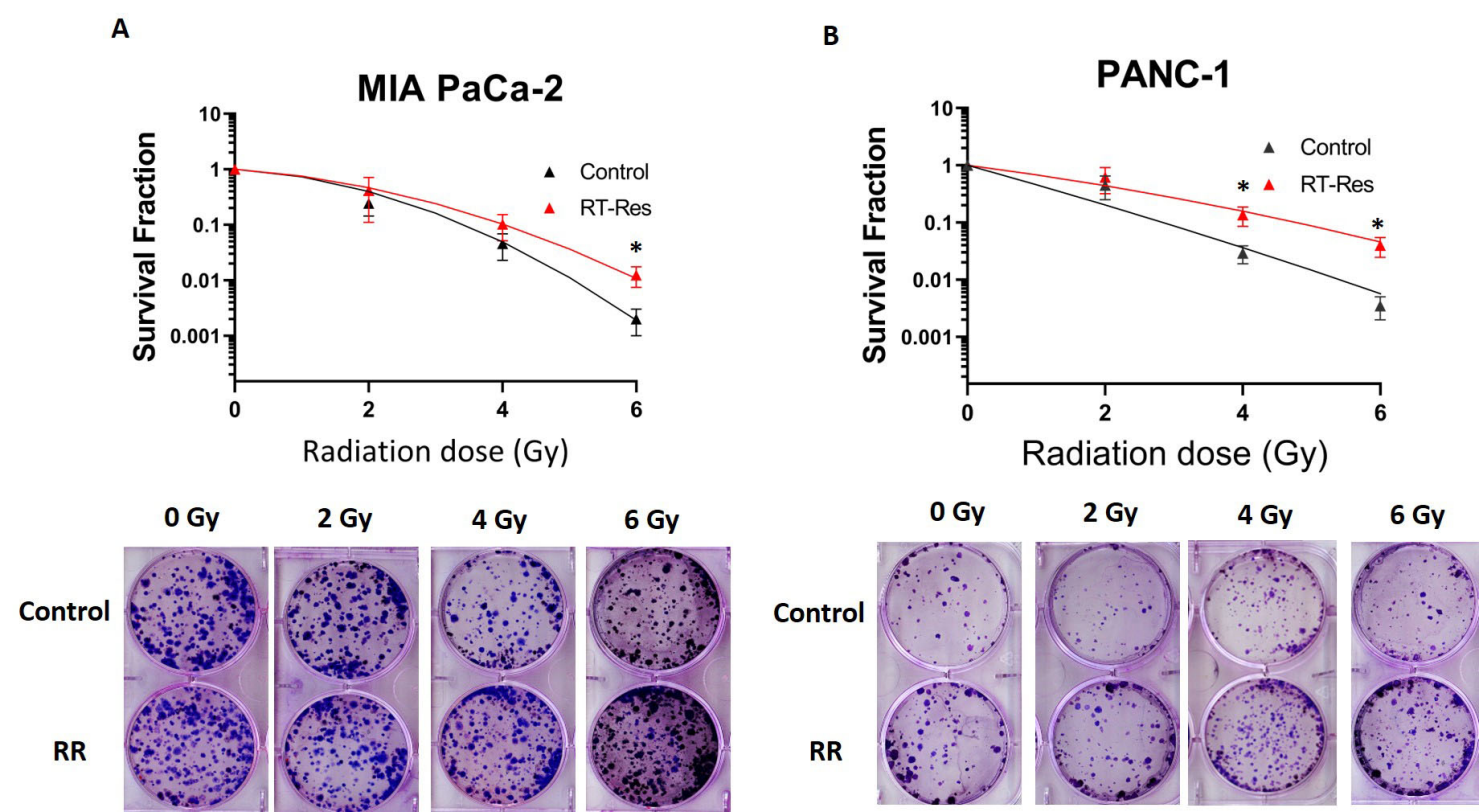
RR PDAC sublines display increased resistance to radiation. Colony formation assay of (A) MIA PACA-2 RR *vs.* MIA PACA-2 and (B) Panc-1 RR *vs.* Panc-1. Representative images of colony formation following increasing doses of IR are displayed below the curves. *P < 0.05; RR: radioresistant.

In both the PDAC cell lines, the generated RR cells showed increased clonogenic survival after exposure to increasing doses of radiation *in vitro *[Figure 2]. For example, at doses of 4 and 6 Gy, the RR cells displayed roughly 50% more clonogenic survival than the parental controls. These clonogenic assay results confirm the increased RR potential of our newly generated PDAC cell lines.

### RNA-seq reveals changes in gene expression following RR

**Figure 3:**
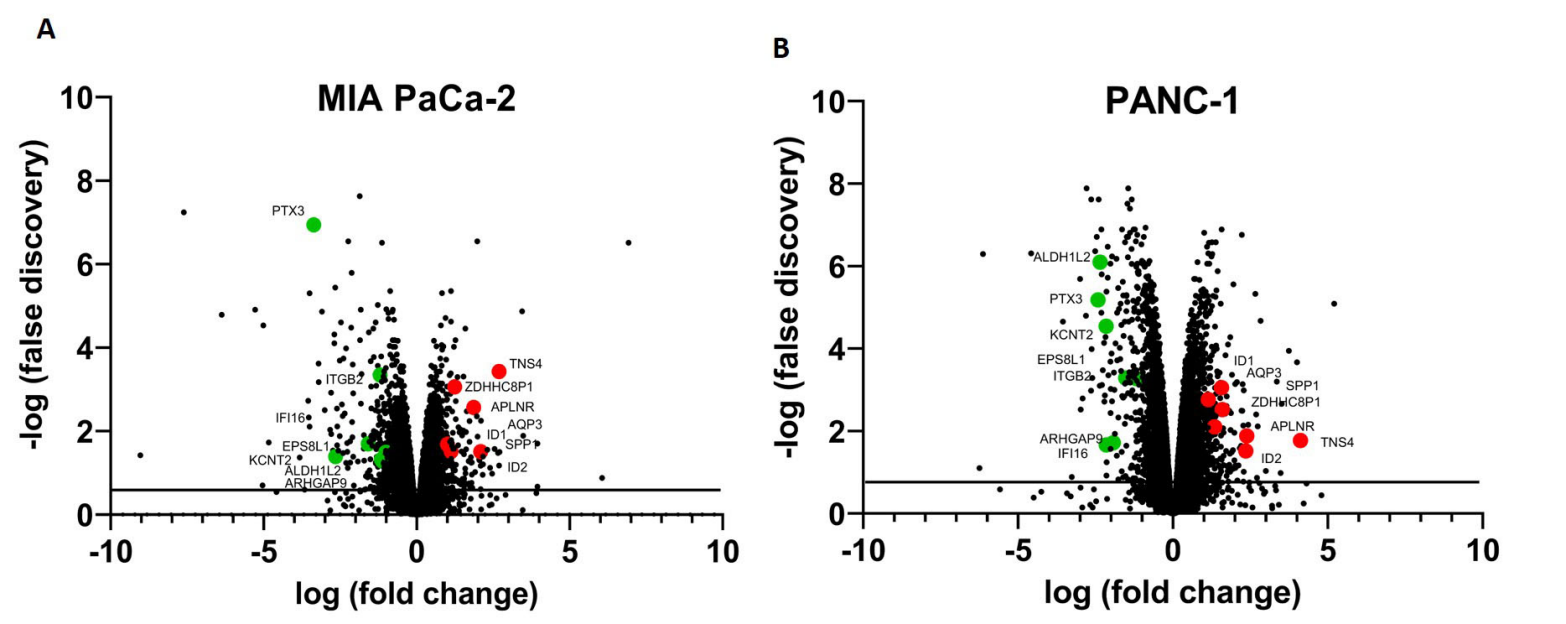
RNA-seq analysis reveals gene expression differences between radioresistant cells and parental cells. Volcano plot representation of differential expression analysis of genes in the parental cells versus RR cells for the MIA PaCa-2 (A) and PANC-1 (B) cells. Red and green points mark the genes with significantly increased or decreased expression in both MIA PaCa-2 and PANC-1 cell lines (FDR < 0.05). The x-axis shows log fold changes in expression, and the y-axis is the negative log of the false discovery value.

RNA-seq analysis comparing global expression levels in the RR cells to parental cells revealed notable differential expression profiles. In the MIA PACA-2 cell line, there was a total of 65 genes found to be upregulated in the RR cells and 136 genes downregulated (adj *P *< 0.05) [Figure 3A]. In the PANC-1 cells, there were a total of 222 genes upregulated in the RR cells and 156 genes downregulated (adj *P *< 0.05) [Figure 3B]. We found a total of 14 genes that were differentially expressed (adj < 0.05) commonly between both the RR cell lines, including seven genes (TNS4, ZDHHC8P1, APLNR, AQP3, SPP1, ID1, ID2) upregulated, and seven genes (PTX3, ITGB2, EPS8L1, ALDH1L2, KCNT2, ARHGAP9, IFI16) that were downregulated.

The full list of genes differentially expressed in both cell lines, function, mutational rate in PDAC, and prior studies examining radiation sensitivity is displayed in Table 1. Most of these genes have some implications for radiation sensitivity, and the gene with multiple studies relating to RR was the ID1 gene. We next analyzed the publicly available cancer cell encyclopedia (CCLE) database, which has IC_50 _treatment response data for the DNA damaging agent 5-Fluorouracil (5-FU) from 600 cancer cell lines including 27 PDAC cell lines. We compared the mRNA expression in the CCLE from each of the significant genes found in our RNA-seq experiment [Supplementary Figures 1 and 2]. We plotted mRNA expression against the IC_50_ value for 5-FU. We found ID1 expression had the highest correlation and strongest *P*-values with higher treatment resistance to 5-FU using Spearman and Pearson correlation tests. These results strengthen our findings that ID1 is a marker for resistance to DNA damaging agents in PDAC. Therefore, we further characterized the ID1 gene as a potential mediator of RR in PDAC patients.

**Table 1:** Significantly upregulated genes in both radiation resistant pancreatic cell lines

**Significantly upregulated genes**				
Gene ID	Gene name	Cellular function*	Mutational frequency in pancreatic cancer^	Association with radiation
APLNR	Apelin receptor	A member of the G protein-coupled receptor gene family. The encoded protein is related to the angiotensin receptor	5.5%	None
AQP3	Aquaporin 3	The water channel protein aquaporin 3. Aquaporin 3 is localized at the basal lateral membranes of collecting duct cells in the kidney	4.6%	AQP3 expression is downregulated by UVA irradiation^[15]^
ID1	Inhibitor Of DNA binding 1	A helix-loop-helix (HLH) protein that can form heterodimers with members of the basic HLH family of transcription factors. The encoded protein has no DNA binding activity and therefore can inhibit the DNA binding and transcriptional activation ability of basic HLH proteins with which it interacts	3.7%	1) In Glioblastoma, PGE2-mediated induction of ID1 is required for optimal tumor cell self-renewal and radiation resistance^[16]^. 2) ID1 overexpression in GBM cells increased radioresistance^[17]^. 3) Id1 and Id3 co-expression seems associated with a poor clinical outcome in patients with locally advanced NSCLC treated with definitive chemoradiotherapy^[18]^. 4) In GBM, a significant correlation (*P* < 0.001) was found between radiotherapy efficacy and ID1 expression levels with respect to overall survival and knockdown of ID1 increased radiosensitivity *in vitro*^[19]^
ID2	Inhibitor Of DNA binding 2	A helix-loop-helix (HLH) protein that can form heterodimers with members of the basic HLH family of transcription factors. The encoded protein has no DNA binding activity and therefore can inhibit the DNA binding and transcriptional activation ability of basic HLH proteins with which it interacts	0.9%	ID2 is induced in response to γ-irradiation^[20]^
SPP1	Secreted phosphoprotein 1	Involved in the attachment of osteoclasts to the mineralized bone matrix	1.8%	SPP1 regulates radiotherapy sensitivity of gastric adenocarcinoma via the Wnt/Beta-Catenin pathway^[21]^
TNS4	Tensin 4	Involved in protein localization. Located in focal adhesion	1.4%	None
ZDHHC8P1	ZDHHC8 pseudogene 1	Pseudogene	NA	None

*https://www.genecards.org/; ^https://www.cbioportal.org/.

### ID1 expression is increased following RT resistance

**Figure 4:**
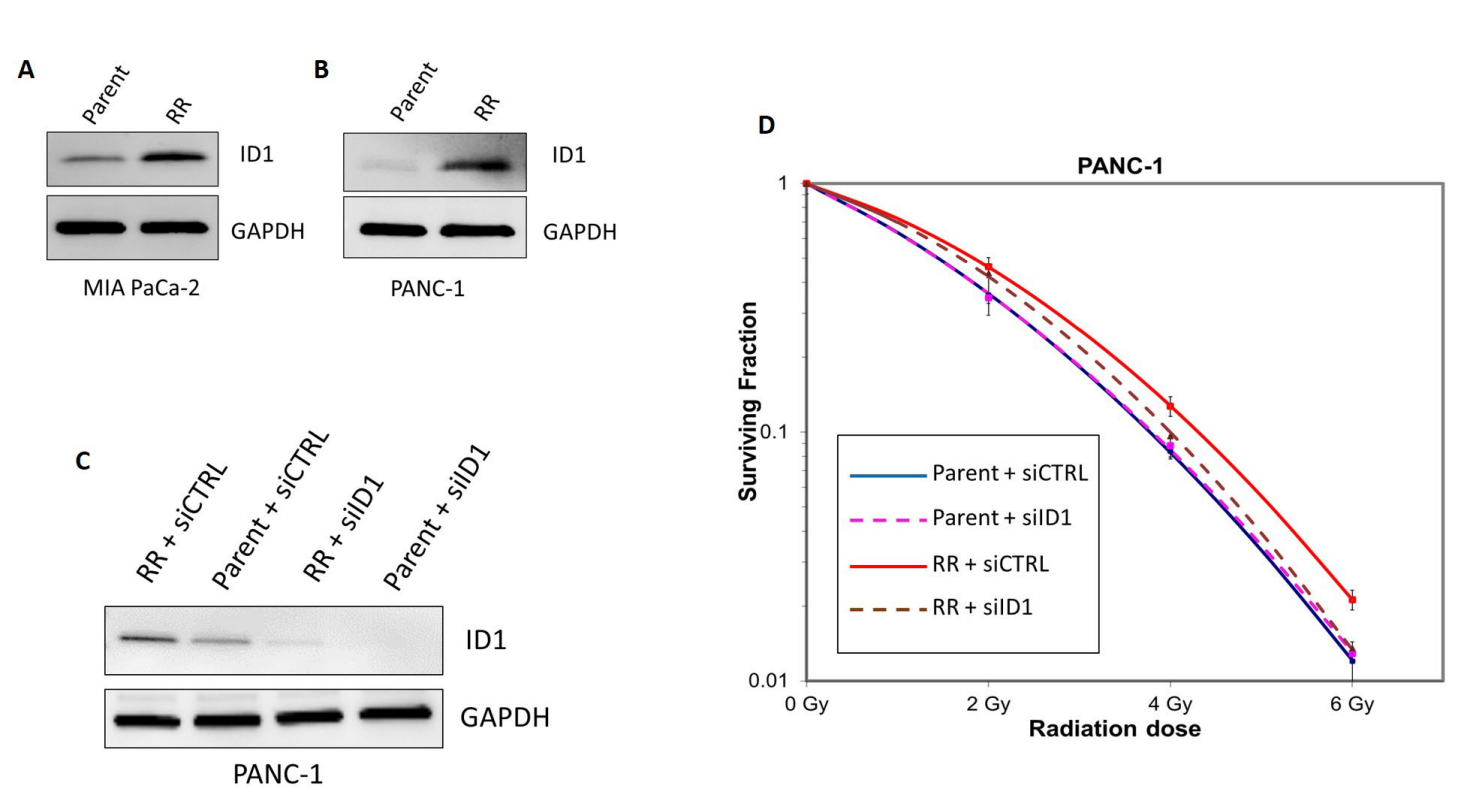
ID1 expression is increased in radioresistant cells. Western blotting confirmed increased protein expression of the ID1 gene in RR cells compared to the parental controls in both (A) MIA PaCa-2 and (B) PANC-1 PDAC cell lines. (C) Western blotting of ID1 and GAPDH 48 h following siRNA transfection of either control or ID1 in parental or RR PANC-1 cells. (D) Clonogenic curves following siControl or siID1 transfection of parent and RR PANC-1 cells. RR: Radioresistant; GAPDH: glyceraldehyde-3-phosphate dehydrogenase.

Following the generation of RR cell lines, we collected the protein lysates of the control and RR cell lines to test the expression of the ID1 protein. Western blotting revealed that ID1 protein expression was increased in the PANC-1 and MIA PACA-2 RR cells compared with parental PANC-1 and MIA PACA-2 cells [Figure 4A and B]. These results confirm the RNA-seq data showing increased ID1 levels in both RR cell lines compared to the parentals. We next sought to address the mechanism of ID1 and radiation resistance. We knocked down the expression of the ID1 protein through siRNA transfection of both the parent and RR PANC-1 cells. After 48 h of siRNA treatment, good knockdown was shown in both cell lines [Figure 4C]. We next performed clonogenic experiments comparing radiation sensitivity in the parent and RR cells following siControl or siID1 pre-treatment. We again show that RR cells display radioresistance compared to the parental cells. We found that siID1 transfection of the RR cells reverted the sensitivity to radiation of the RR cells compared to the siControl parental cells. There was no impact on the radiation sensitivity of the parental cells following siID1 transfection [Figure 4D]. These results suggest that ID1 is activated in the radiation resistance population, and targeting ID1 could be a strategy to increase sensitivity to radiation.

### ID1 is a potential biomarker in PDAC patients

**Figure 5:**
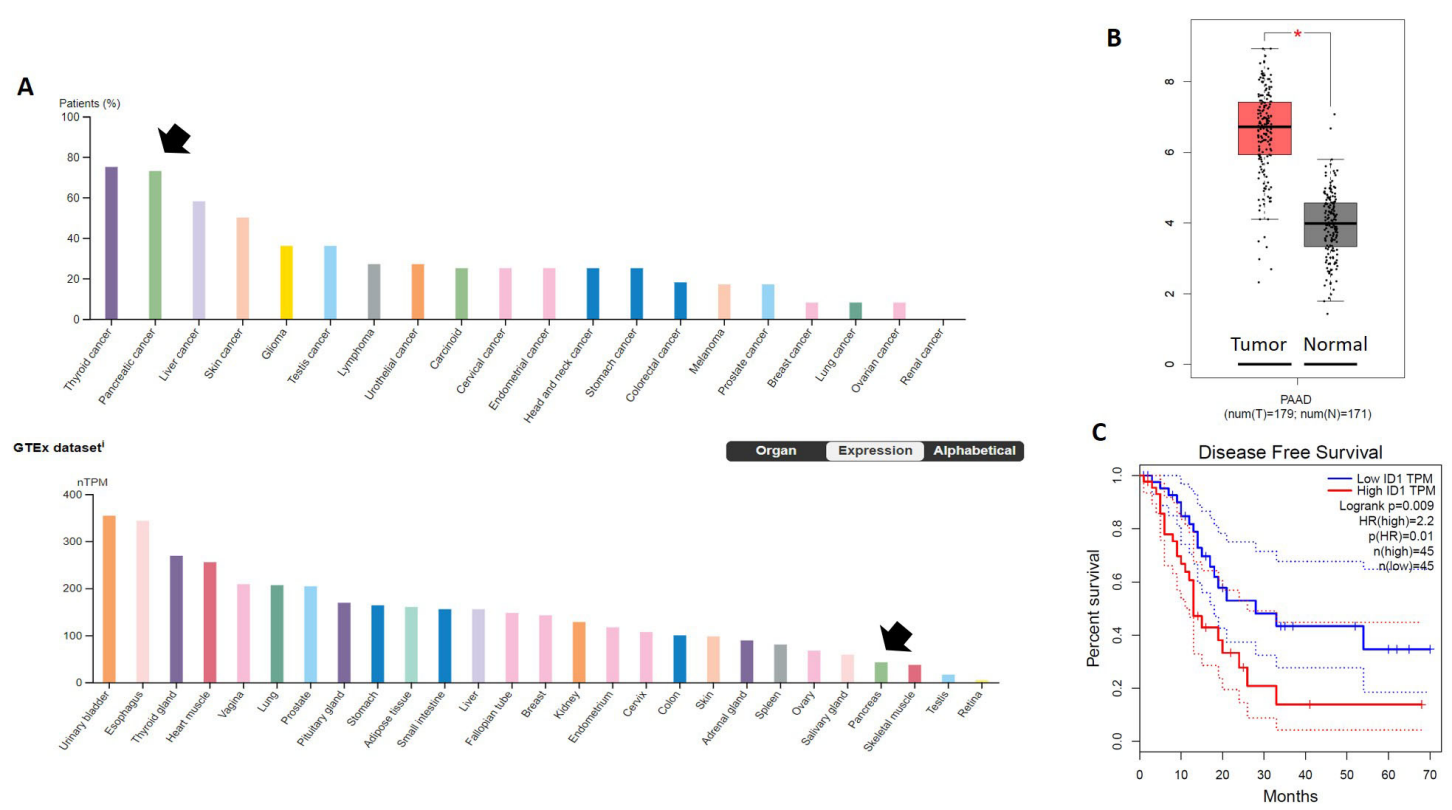
ID1 expression is high in PDAC tissue compared to normal samples and correlates with worse disease survival. (A) The percent of tumor samples with an either high or medium expression of ID1 on immunohistochemistry (IHC) was rank-ordered. The mRNA expression of ID1 in normal tissue was rank-ordered in normal tissue samples from the GTEx RNA-seq dataset. Arrow points to PDAC and pancreas tissue. (B) Direct comparison of mRNA expression data from PDAC tumors in the TCGA and normal pancreatic tissue from GETx. *P < 0.05. (C) PDAC patients in the TCGA dataset were grouped based on mRNA expression of ID1 split on the median value. High ID1 tumor expression predicted worse disease-free survival, *P *= 0.009 HR = 2.2.

To evaluate the expression profile of ID1 in PDAC, we analyzed the available tissue sample data from both IHC and RNA-seq data from PDAC patients in the TCGA project and normal samples in the GTEx project using the human protein atlas website. As shown in Figure 5A, ID1 high or medium protein expression was found in 75% of pancreatic cancer patients, and PDAC was the second-highest ID1 expressing cancer behind only thyroid cancer. In the normal tissue samples from the GTEx RNA-seq dataset, the normal pancreas had relatively low expression compared to other tissues. The pancreas was the 4th lowest normal tissue expression [Figure 5A]. The direct comparison of ID1 mRNA expression in PDAC tissue *vs.* normal pancreas tissue showed significantly higher ID1 expression in the tumor samples compared to normal tissues (*P *< 0.05) [Figure 5B].

We next investigated the association between ID1 expression and disease-free survival (DFS) from the TCGA database. Survival analysis using Kaplan-Meier and log-rank test divided at the median value revealed lower DFS in PDAC patients with high ID1 expression compared to patients with low ID1 expression [hazard ratio (HR) = 2.2, *P *= 0.009] [Figure 5C].

**Figure 6:**
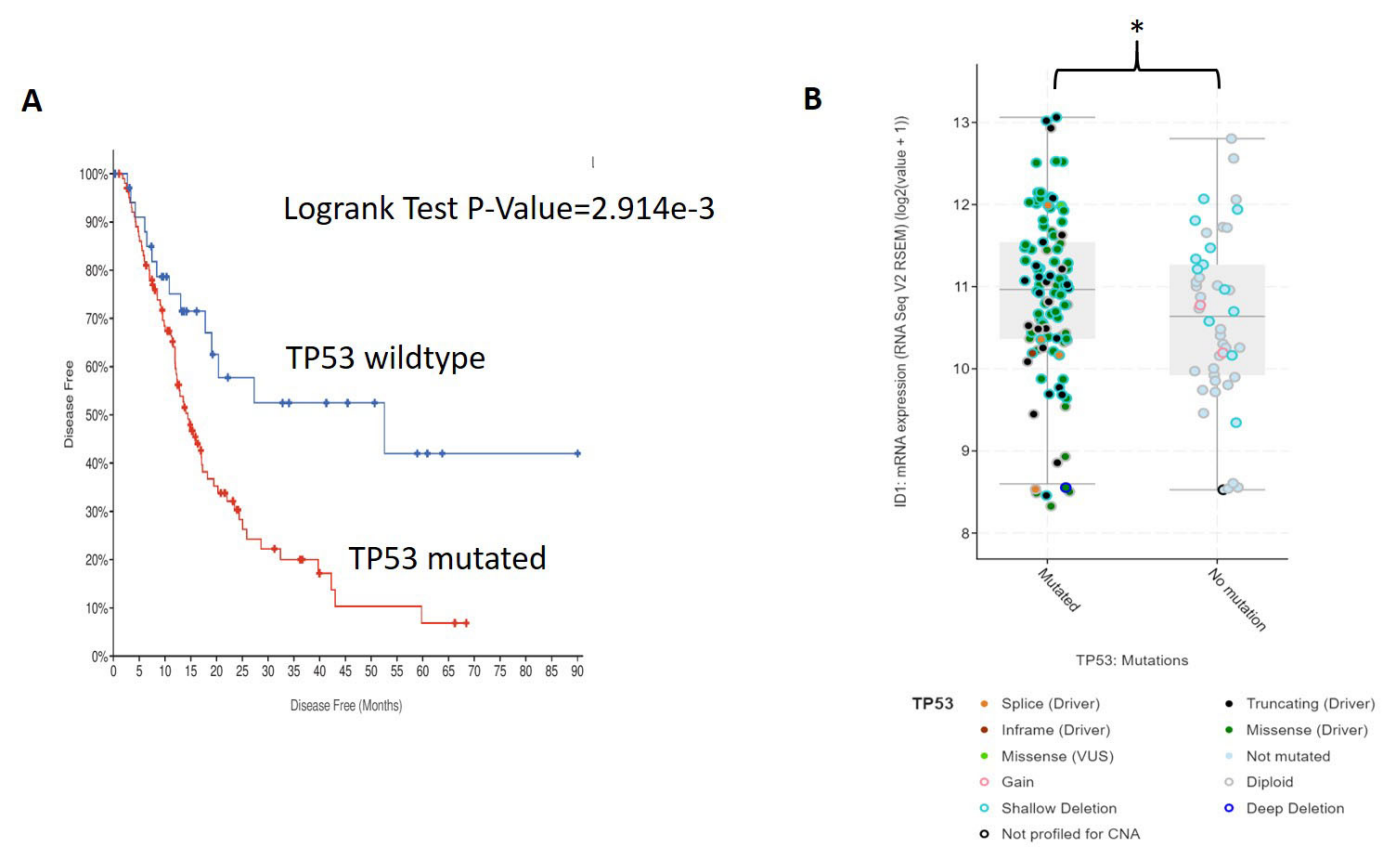
TP53 mutation is a poor prognostic factor and correlates with higher ID1 expression. (A) PDAC patients in the TCGA dataset with tumor p53 mutation (63%) were compared to p53 wildtype for disease free-survival (DFS). Patients with p53 mutations had significantly worse DFS than p53 wildtype, *P *= 0.003. (B) ID1 mRNA expression was compared in p53 mutated tumors *vs.* p53 wildtype tumors from the TCGA PDAC dataset. ID1 expression was higher in the p53 mutated patients *P < 0.05.

The p53 gene is a known regulator of cellular response to radiation therapy^[22]^. Patients with mutated p53 in multiple cancer types, including PDAC, have worse RT response rates than p53 wildtype patients^[23]^. From our analysis of the TCGA PDAC cohort, p53 mutant patients had lower DFS than p53 wildtype patients (log rank *P *= 2.9e-3) [Figure 6A]. We next sought to determine if there was a correlation between p53 mutation and ID1 expression. In the TCGA tumor dataset, we found that p53 mutant PDAC patients had significantly higher ID1 mRNA expression than p53 wildtype patients (*P *= 0.03) [Figure 6B].

Together, our data showed that expression of ID1is upregulated in PDAC and is associated with poor survival and p53 mutation, supporting ID1 as a potential mediator in PDAC RR.

## DISCUSSION

Although the development of metastatic disease dominates the natural history of PDAC, local tumor progression contributes significantly to morbidity and mortality. Conventional RT (cRT) with small 1.8-2 Gy/fraction doses has shown low long-term tumor control rates or survival benefits. Over the past decade, dose-escalation strategies (otherwise termed ablative RT, or aRT (3-4 Gy/fraction)), have shown promising results^[24-27]^. The aRT technique requires a highly specialized team of Radiation Oncologists and Physicists in addition to state-of-the-art technologies such as daily adaptive MR linear accelerators, only available in a handful of centers in the U.S.A. Outside of large tertiary cancer centers, delivery of aRT while safely avoiding normal tissue dosing is possible for a minority of PDAC patients where tumors are far enough away from the luminal gastrointestinal (GI) tract. Therefore, novel strategies to improve radiosensitivity to standard lower doses of RT are critical. To enhance radiosensitivity, genetic drivers of RR are crucial to be discovered to develop novel targeted therapeutics to combine with RT.

Two previous studies have studied gene regulation of radiation resistance using engineered radioresistance PDAC cell lines. Souchek *et al.*^[28] ^and Ogawa *et al.*^[29]^ used the cDNA microarray method to identify gene changes following radioresistance. The Soucheck study found the cholesterol pathway to be significantly upregulated in radioresistance cells, including genes: FDPS, ACAT2, AG2, CLDN7, DHCR7, ELFN2, FASN, SC4MOL, SIX6, SLC12A2, and SQLE. The Ogawa study found upregulated pathways related to growth factors, cell cycle checkpoint, and angiogenesis, including genes: AREG, MAPKAPK2, RGN, ANG-2. Our study generated RR PDAC cells and utilized the RNA-seq to further increase the current understanding of gene expression changes in radioresistant PDAC cell populations. The novelty of our study is that we used a clinically relevant dose fractionation of 2 Gy/day to generate our RR cell lines compared to a single 10 Gy dose in the Ogawa study. The Soucheck study also used this clinically relevant dose fractionation, but in contrast to our study, it only utilized one cell line. In this study, two different PDAC cell lines were generated, and genes that were upregulated in both were analyzed further, increasing the robustness of the current study.

Other studies have examined intrinsic radiation sensitivities in various PDAC cell lines to study potential therapeutic vulnerabilities. Wiechmann *et al.* screened 38 PDAC cell lines and pulled out two radiation sensitive and two resistant cell lines to study the phosphoproteomes in response to 8 Gy of radiation^[30]^. They found increased actin dynamics and FAK activity in the resistant cell lines and FAK inhibition radiosensitized radioresistant cell lines more than the radiosensitive cell lines. Schröter *et al.* examined the role of high dose radiation and changes in cell surface expression of immunomodulatory molecules^[31]^. Interestingly, they found that only high radiation doses of > 5 Gy increased surface expression of PD-L1 and CD73. These results have implications for the potential use of immunotherapy combinations with radiation such as stereotactic body radiotherapy (SBRT) which utilizes high doses per fraction of radiation.

In our study, we generated new RR PDAC cell lines and then further characterized the changes in gene expression using next-generation-based RNA-Seq. The new cell lines displayed higher survival levels after radiation *in vitro*, and we discovered 14 genes that had significant changes in expression in the RR cell lines compared to the parental. Of these genes, we explored one gene, ID1, that has multiple prior published reports in other cancer types related to RR.

ID1 is a helix-loop-helix (HLH) protein that can form heterodimers with members of the basic HLH family of transcription factors. The encoded protein has no DNA binding activity and, therefore, can inhibit the DNA binding and transcriptional activation ability of essential HLH proteins with which it interacts^[32]^. Maruyama *et al.* reported that PDAC cells expressed high levels of the ID genes and that ID1 expression was increased compared to normal controls. The authors concluded that increased ID1 expression might be associated with the enhanced proliferative potential of pancreatic cancer cells^[33]^. In our analysis of the TCGA database, we found that ID1 expression was highly expressed in pancreatic tumor samples compared to normal pancreas tissue [Figure 5].

There have been several prior published publications examining the role of ID1 in radiation resistance, reviewed in Table 1. Cook *et al.* showed that PGE2 signaling regulated radiation resistance in mouse glioblastoma (GBM) primary cultures in an ID1-dependent manner^[16]^. In GBM, ID1 overexpression increased radioresistance^[17]^, and ID1 expression levels predicted poor overall survival and knockdown of ID1 increased radiosensitivity *in vitro*^[18]^. One of the other genes from our RNA-seq data shown to regulate radiation sensitivity was SPP1 in gastric adenocarcinoma^[21]^.

To explain the potential role of ID1 in associating with RR in PDAC cells, we found that patients with p53 mutation had significantly higher expression of ID1 mRNA than p53 wildtype patients. Qian and Chen reported that ID1 expression was downregulated following DNA damage in a p53-dependent manner. They found that ID1 overexpression promoted cell proliferation and inhibited DNA damage-induced cell senescence. They concluded that ID1 was a critical p53-dependent DNA damage response pathway^[34]^.

Looking into the future, ID1 may serve as a therapeutic target to increase the radiosensitivity of PDAC tumors and improve the outcomes in PDAC. Further studies developing strategies to target ID1 are warranted.

## DECLARATIONS

### Availability of data and materials

All models are available to academic centers under an institutional MTA.

### Copyright

© The Author(s) 2022.
