# Supplementary material for: Discovery of the inhibitor of DNA binding 1 as a novel marker for radioresistance in pancreatic cancer using genome-wide RNA-seq
Source: Cancer Drug Resist. 2022 Oct 18;5(4):926–38. doi: 10.20517/cdr.2022.60 (PMC9771737; doi:10.20517/cdr.2022.60)
Supplement: Supplementary file 1 [file cdr-5-4-926-SupplementaryMaterials.pdf]

Supplementary Figure 1

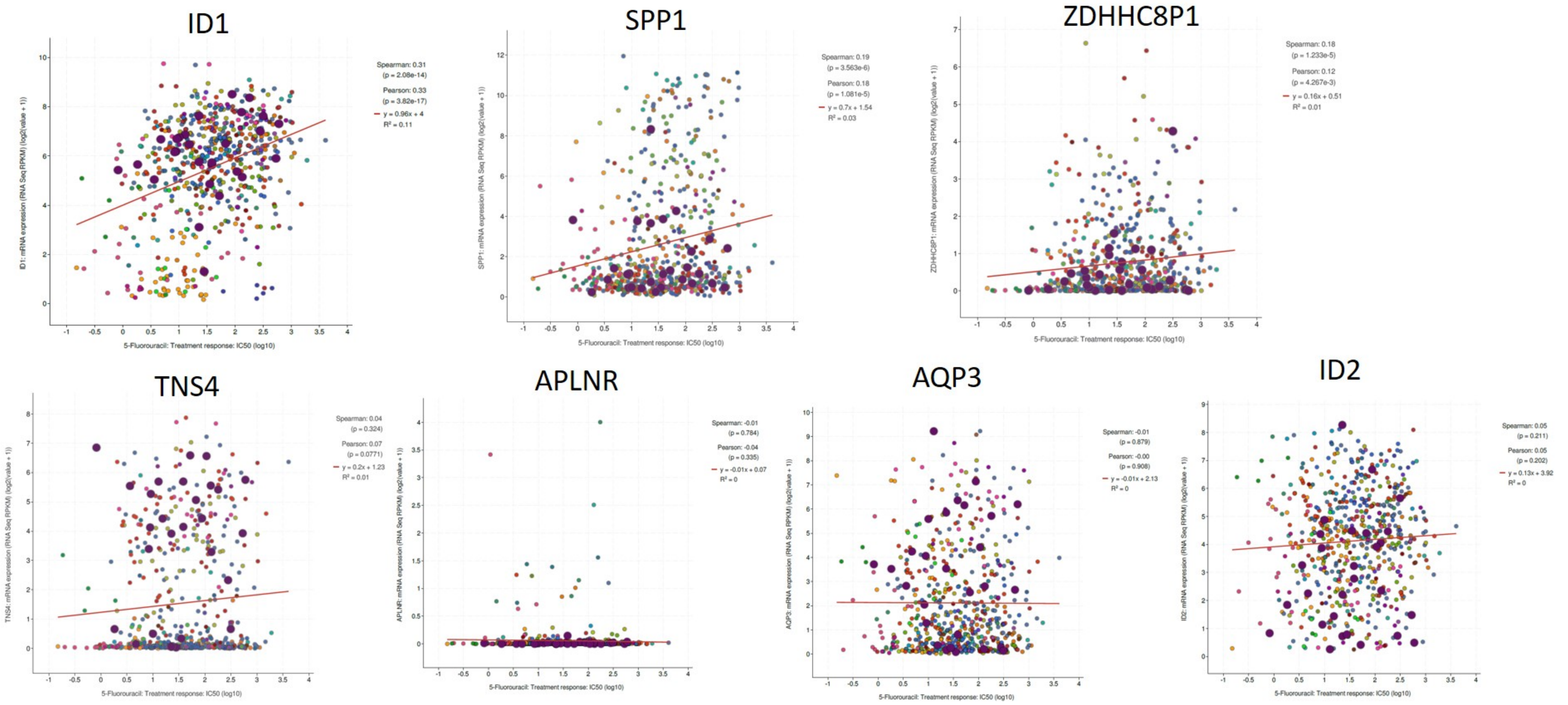

Significantly upregulated gene mRNA expression in the RR cell lines compared to sensitivity to 5-Fluorouracil chemotherapy in the cancer cell lines available in the CCLE database. Gene expression on the y-axis and IC50 5-FU on the x-axis. Large purple circles represent pancreatic cancer cell lines. Graphs generated no https://www.cbioportal.org/

Supplementary Figure 2

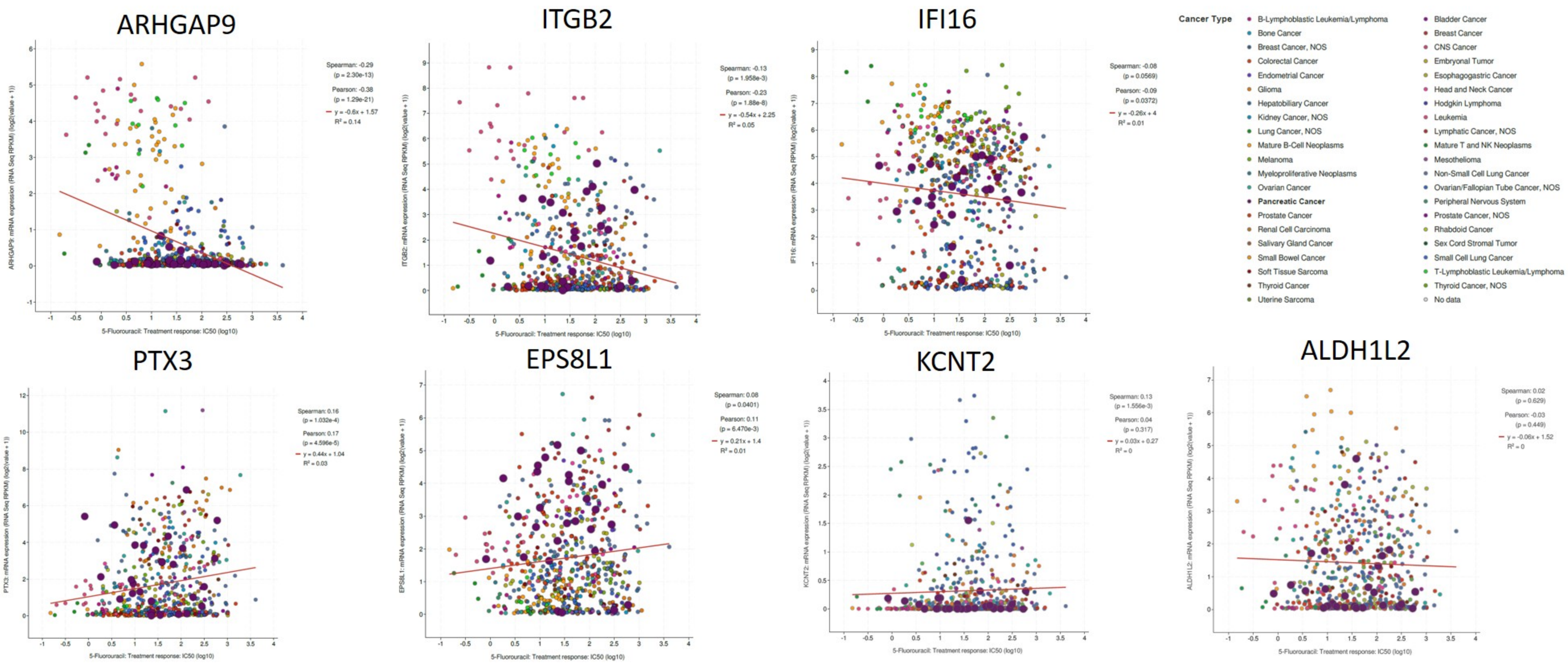

Significantly downregulated gene mRNA expression in the RR cell lines compared to sensitivity to 5-Fluorouracil chemotherapy in the cancer cell lines available in the CCLE database. Gene expression on the y-axis and IC50 5-FU on the x-axis. Large purple circles represent pancreatic cancer cell lines. Graphs generated no https://www.cbioportal.org/
